# An Open-Source Wireless Electrophysiological Complex for In Vivo Recording Neuronal Activity in the Rodent’s Brain

**DOI:** 10.3390/s21217189

**Published:** 2021-10-29

**Authors:** Alexander Erofeev, Dmitriy Kazakov, Nikita Makarevich, Anastasia Bolshakova, Evgenii Gerasimov, Arseniy Nekrasov, Alexey Kazakin, Ivan Komarevtsev, Marina Bolsunovskaja, Ilya Bezprozvanny, Olga Vlasova

**Affiliations:** 1 Laboratory of Molecular Neurodegeneration, Graduate School of Biomedical Systems and Technologies, Institute of Biomedical Systems and Biotechnology, Peter the Great St. Petersburg Polytechnic University, 195251 Saint Petersburg, Russia; asyab@mail.ru (A.B.); evgeniigerasimov1997@gmail.com (E.G.); mnlabspb@gmail.com (I.B.); 2 National Technology Initiative Center for Advanced Manufacturing Technologies, Laboratory of Industrial Data Streaming Systems, Peter the Great St. Petersburg Polytechnic University, 195251 Saint Petersburg, Russia; dmitriy.kazakov@spbpu.com (D.K.); makarevich.98@mail.ru (N.M.); bolsun_mv@spbstu.ru (M.B.); 3 Neuropribor, Limited Liability Company, 194223 Saint Petersburg, Russia; hypernyan@gmail.com; 4 Laboratory of Nano- and Microsystem Technology, Joint Institute of Science and Technology, Peter the Great St. Petersburg Polytechnic University, 195251 Saint Petersburg, Russia; Kazakin75@gmail.com (A.K.); imk@spbstu.ru (I.K.); 5 Department of Physiology, University of Texas Southwestern Medical Center at Dallas, Dallas, TX 75390, USA

**Keywords:** wireless wearable module, printed circuit board, base charging station, electrical circuit diagram, software, neuronal activity, rodent’s brain, multi-electrode array, electrophysiological complex

## Abstract

Multi-electrode arrays (MEAs) are a widely used tool for recording neuronal activity both in vitro/ex vivo and in vivo experiments. In the last decade, researchers have increasingly used MEAs on rodents in vivo. To increase the availability and usability of MEAs, we have created an open-source wireless electrophysiological complex. The complex is scalable, recording the activity of neurons in the brain of rodents during their behavior. Schematic diagrams and a list of necessary components for the fabrication of a wireless electrophysiological complex, consisting of a base charging station and wireless wearable modules, are presented.

## 1. Introduction

One of the trends in modern brain research is a general global trend of finding the most informative ways to register and analyze neuronal activity. For this reason, the multi-electrode arrays (MEAs) are actively implemented into experiments in vitro/ex vivo [1,2] and in vivo on freely moving animals [3,4,5,6,7,8,9,10,11]. The electrodes in a multi-electrode array transduce the change in voltage from the environment carried by ions into currents carried by electrons. The voltage arises due to the transmembrane currents of neurons and is an integral measure of the activity of a part of the nervous tissue.

Multi-electrode arrays have their advantages and disadvantages as a method of recording neuronal activity. The main advantages of multi-electrode arrays for in vitro and ex vivo experiments are the simultaneous registration of a large amount of data, analysis of the activity of neural networks, and drug testing. Additionally, using MEAs for cell cultures does not violate the integrity of the cell membrane in comparison with the patch clamp (whole-cell). In experiments in vivo, MEAs make it possible to register neural activity in the target areas of the brain during the animal behavior and detect changes in neural correlations [12,13,14]. For this, a microelectrode array is implanted into the animal’s brain, in accordance with the stereotaxic atlas. Electrodes are sized to minimize invasiveness and register the biopotential of several hundred nearby neurons of the target brain region of the experimental animal [8]. The disadvantages of using MEAs for registration and stimulation of individual cells in vitro/ex vivo is their low spatial resolution in comparison with patch-clamp. In the case of the in vivo application of MEAs, the disadvantages will be the loss of neuronal cells, scarring of glia and a decrease in the number of working electrodes due to their gradual overgrowth with connective tissue.

Despite the disadvantages of multi-electrode arrays, they are widely used in modern neurobiology. In recent years, attempts have been made to combine multi-electrode arrays with other methods of recording or influencing neural activity, for example, using optogenetics [15] or a miniscope [16]. In addition, research is underway in the field of biocompatible polymers [17,18,19,20] and flexible MEAs [21,22,23,24,25] to address the problems associated with the loss of neuronal cells, scarring of glia and a reduction in the number of working electrodes. Research is also underway to improve algorithms for processing the data obtained using multi-electrode arrays [26,27]. Thus, it can be concluded that, despite their shortcomings, multi-electrode arrays are widely used in modern research and are a promising tool for researchers.

Nevertheless, the main efforts of researchers are aimed at improving and finalizing multi-electrode arrays [15,17,21,28,29] or software for MEAs and electrophysiological recordings [28,29,30,31,32,33,34,35,36,37] and are much less often presented with ready-made solutions for recording and processing data from a multi-electrode array [38,39,40,41,42,43]. Data acquisition systems on the market and in scientific research are expensive or difficult to create. For this reason, an inexpensive scalable solution becomes relevant. In this article, we propose a ready-made open-source solution that can be modified to suit the needs of researchers: an electrophysiological complex, which is a system for collecting and visualizing data in real time and saving them in csv format for further processing and analysis. To do this, we analyzed the solutions existing on the market (Table 1). Based on the results of the analysis, the following characteristics are formulated:

1. Small size and weight (15 × 10 × 10 mm; ~2 g);
2. The possibility of using a multi-electrode array with a different number of recording channels;
3. The ability to measure the impedance of electrodes;
4. The ability to perform electrical stimulation;
5. The possibility of using in optogenetics experiments (light stimulation);
6. Wireless signal transmission;
7. High data transfer rate (1 Mbps);
8. Long battery life (at least 3 h).

In most cases, the multi-electrode array is connected to the data collection, processing and transmission unit via a cable. The use of the cable imposes a number of restrictions on the use of the MEAs in behavioral tests due to problems with twisting and fixed cable length. In this regard, a number of compact wireless multi-electrode arrays have been developed, which are used in neuronal activity studies [10]. The wireless version, on the one hand, has an obvious advantage over the wired version, and on the other hand, it has a significant drawback in the form of the obligatory presence of a heavy lithium-polymer power source (~1.1 g). We believe that, despite the above disadvantages, the future of multi-electrode arrays lies precisely with wireless technologies. For this reason, we have made the electrophysiological complex wireless.

As a result, an open-source wireless electrophysiological complex (WEC) was created for recording neuronal activity in the rodent’s brain.

In the current version of the WEC, the following are not implemented: the ability to perform electrical and light stimulation. We plan to implement these functions in future versions of the WEC.

We chose to implement an electrophysiological complex comprising several parts: **wireless wearable module with battery (wireless module)** and **base charging station (base station)**. A wireless module is a wearable rodent unit. This module includes a charge with a special connector for charging and storage in the base station. The multi-electrode array will be connected to a wireless module, which will be responsible for registering neural activity in the rodent’s brain and sending data to the base station. The base station is required for receiving and transmitting the signal, as well as charging the wireless modules.

**Figure 1:**
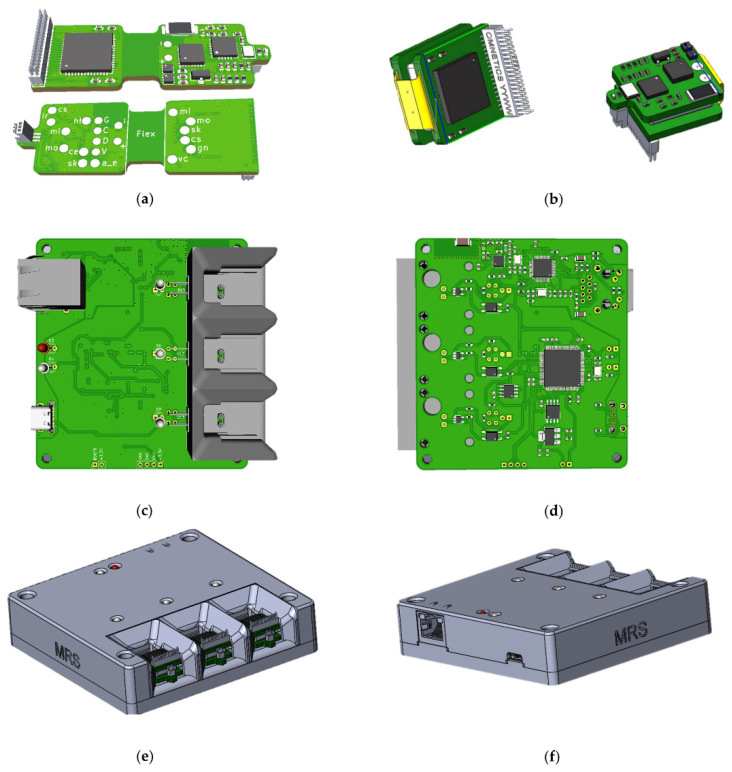
Printed circuit boards of wireless module: (**a**) top view and bottom view, (**b**) folded and base station: (**c**) top view, (**d**) bottom view; 3d model of base station: (**e**) front sides, (**f**) back sides, housing.

In the process of creating the wireless module and the base station, attention was paid to ergonomic issues, and the following issues were also resolved: how to charge the wireless modules, how to manage their power supply, how to store and transport the entire product, how many modules should be included in one base station, etc. As a result, 3D models of printed circuit boards and housing of the base station (Figure 1) were created, according to which prototypes were subsequently made.

The components of the WEC are discussed in more detail in the following sections of the article. The list of required components, required files, and software can be found in the GitHub repository at the link—https://github.com/Neuropribor/wec (accessed on 19 October 2021).

## 2. Materials and Methods

### 2.1. Multi-Electrode Array

**Figure 2:**
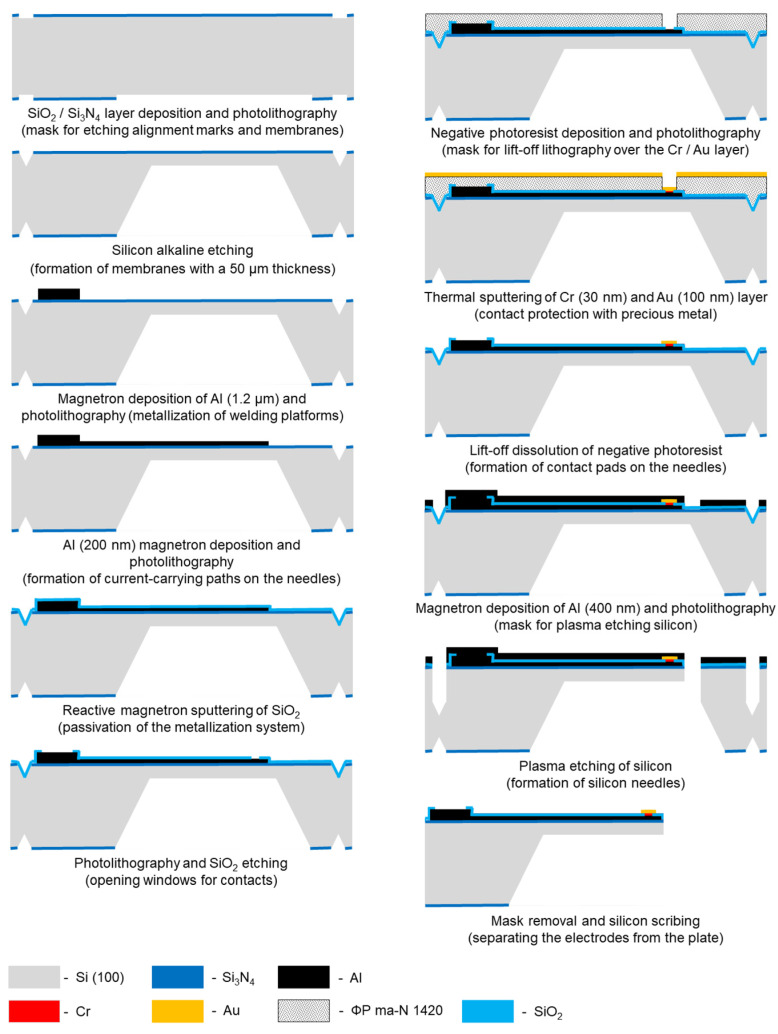
Technological route for the manufacture of multi-electrode array.

A multi-electrode array was created using photomasks for contact photolithography when creating a system of multilevel metallization, passivation, and shaping needles. Multilevel metallization was carried out by the thermal deposition of films of aluminum 1200 nm and 200 nm thick, chromium (30 nm), gold (100 nm). Lift-off photolithography was performed on these films using a negative photoresist ma-N 1420. Silicon oxides of 200–300 nm thick, deposited by reactive magnetron sputtering, were used as the dielectric. The needles of the multi-electrode array are formed by vertical plasma etching of silicon (RIE); the formation of a plasma-resistant mask was realized on a layer of aluminum with a thickness of 400 nm. Individual electrodes were prepared after wet removal of the mask and diamond disc-cutting of the wafer. The electrodes were mounted on the board by welding with gold conductors using ultrasonic welding technology. The complete technological route for the manufacture of electrodes is shown in Figure 2.

### 2.2. Wireless Module, Base Station, and Software

The PCB layouts for the wireless module and base station were created in open-source software KiCad EDA version 5.1.10 and then sent for production. The manufacturing of printed circuit boards and components installation on PCBs of the wireless modules was carried out at the PCBWay Factory (pcbway.com, Shenzhen, China). The manufacturing of printed circuit board and components installation on PCB of base station was carried out at the JLCPCB Factory (jlcpcb.com, Shenzhen, China). A schematic diagram and layouts of the printed circuit boards can be found in Appendix A. For the wireless module and base station, special housings with fastening have been developed. The design of the enclosures and 3D models of the wireless module and base station were created using SolidWorks 2016 (Dassault Systèmes, Vélizy-Villacoublay, France). SLA photopolymer 3D printing was chosen for the fabrication of the wireless module housings due to its manufacturing accuracy. Printing of wireless modules was carried out at a scale of 1:1 on a Formlabs Form 2 printer (Formlabs, Somerville, MA, USA), durable resin photopolymer (Formlabs, Somerville, MA, USA). The base station housing was printed on a FDM printer with a 0.3 mm layer of PLA plastic and a nozzle with a diameter of 0.5 mm.

### 2.3. Firmware

**Figure 3:**
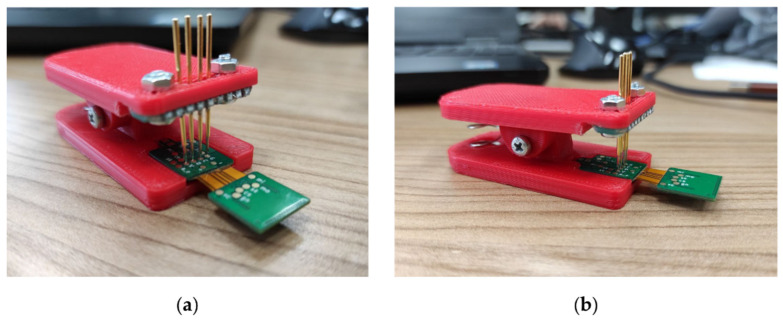
Clip adapter with spring-loaded contacts: (**a**) front view, (**b**) side view.

Firmware and data visualization software were written for the wireless module and base station. Wireless module firmware is designed to configure the analog-to-digital converter, read data, process the data transfer protocol, transmit to the base station, control the operating modes and switch to power-saving mode. Base station firmware performs configuration and operation with the radio module, communication via Ethernet, interaction with the upper-level software, and the formation of a continuous data stream from wireless modules to the PC. For the convenience of flashing and debugging the firmware of wireless modules, a clip with spring-loaded contacts was developed (Figure 3).

Firmware of wireless module and base station was written in bare-metal C language and C language with RTOS FreeRTOS and LwIP stack, respectively. Firmware was created in the STM32CubeIDE program (STMicroelectronics, Geneva, Switzerland) and flashing through the swd interface using the ST-Link V2 programmer.

### 2.4. Data Visualization and Recording Software

Modern approaches to software development primarily aim to reducing the complexity and reuse software modules. This can be achieved through the use of high-level programming languages. For data visualization and recording software, we used Python 3, a Socket interface for interacting with Ethernet. PyQt5 for GUI and PyQtGraph was used for drawing graphs in real-time mode.

## 3. Results

### 3.1. Multi-Electrode Array

**Figure 4:**
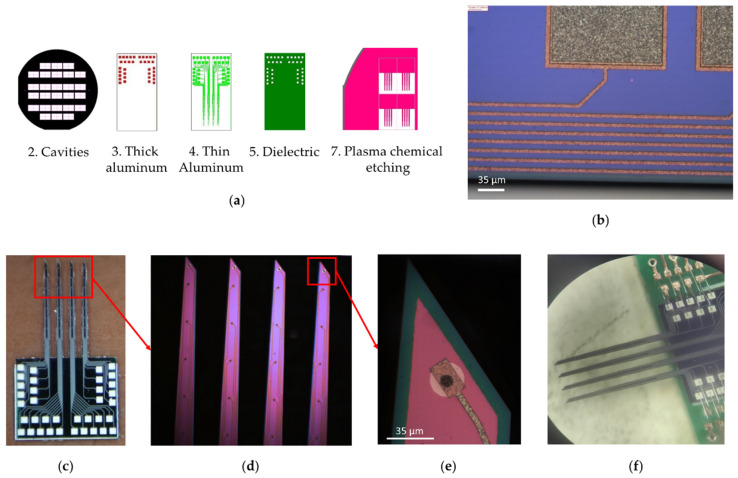
Multi-electrode array. (**a**) Photomasks with drawings of functional layers of a multi-electrode array; (**b**) Elements of two-level aluminum metallization (matt gray is contact pads; light brown is conductive tracks); (**c**) 36-channel electrode wafer; (**d**) Enlarged view of MEA silicon needles (4 needles, 5 mm long, 260 μm average width and 50 μm thick); (**e**) Enlarged view of MEA needle tip (green represents first layer of insulating silicon dioxide, red represents second layer of insulating silicon dioxide, white represents gold contacts, brown represents aluminum pads, black represents electrical connection between golden and aluminum pads); (**f**) 32-channel multi-electrode array soldered with gold on a special board, one channel on each of the four needles was used to control the conductivity of the MEA tracks.

To create a multi-electrode array, a set of seven photomasks with drawings of functional layers of electrodes was made; examples of the topology of some layers are shown in Figure 4. Template 1 “Alignment marks” was used to form two-sided alignment marks on the front and back sides of the silicon wafer. Template 2 “Cavities” was used to form a mask for the deep alkaline etching of silicon from the backside of the wafer. Template 3 “Thick aluminum” was used to form the contact elements of metallization on the welding pads of the electrodes. Template 4 “Thin Aluminum” was used to form the current-carrying tracks of the needle. Template 5 “Dielectric” was used to open windows in a dielectric above the contact pads. Template 6 “Contacts”, with a negative pattern, was used to form gold contact pads on the tip of the needles. Template 7 “PCE” was used to form a mask for the plasma etching of silicon needles. The designs of photomasks 1 and 6 are not shown because their individual elements, with a width of 20 µm, are indistinguishable against the background of both the whole wafer and separate chips.

The multilevel metallization system was implemented as follows: Al (1200 nm) → Al (200 nm) → Cr (30 nm) → Au (100 nm). Individual elements are shown in Figure 4b. The first layer of aluminum on the welding pads has a thickness of 1.2 µm. Elements of 300 × 300 and 300 × 600 µm in size were formed from this for the subsequent ultrasonic welding of wires at the stage of mounting electrodes (Figure 4b, matte gray color). The second layer of aluminum was 200 nm thick. From this, current-carrying paths were attached to the contact pads located on the tips of the needles (Figure 4b, light brown). The width of the tracks was 10 µm; the minimum distance between them was 15 µm. The entire surface of the aluminum metallization was covered with a protective dielectric layer, except for the points of contact with brain neurons and areas for welding wires. Reactively deposited silicon oxide with a thickness of 200–300 nm was used as a dielectric.

The silicon surface (400 μm) of multi-electrode array was covered with an insulating silicon dioxide layer (green, Figure 4e). Along each needle, at equal distances, there were eight round gold contacts 100 nm thick with a diameter of 15 μm (white, Figure 4e), which were electrically connected to the aluminum pads using metallization tracks (brown, Figure 4e) for switching with the measuring board (silver, Figure 4c). The electrical connection of gold contacts and aluminum tracks was carried out through windows with a diameter of 10 μm etched in silicon dioxide (black, Figure 4e). A layer of chromium 30 nm thick was applied between the aluminum and gold to prevent the destruction of the contact pads due to the formation of intermetallic compounds. An additional 9-th channel with a gold area of 25 μm in diameter was used to control the conductivity of the tracks during the manufacture of the electrode. The distance between the first and second contact pads, starting from the tip of the needle, was 247 μm and 500 μm between the rest of the contact pads. The entire metallization system, except for the gold contacts and aluminum switching pads, was covered with a second layer of silicon dioxide (red, Figure 4e).

The multi-electrode array needles (5 mm long, 260 μm average width and 50 μm thick, Figure 4d) were formed by vertical reactive ion etching (RIE) at a depth of 60 µm for a silicon wafer with a 400 nm thick aluminum mask. Such a mask can withstand intense heating and ion bombardment during the RIE process, and can also be selectively removed later without destroying other materials on the surface of the electrodes. The heat sink contained a vacuum oil layer under the silicon wafer.

After removing the mask, the wafer was divided into separate electrodes by the method of diamond disk cutting. As a result, a wafer of a separate electrode was made, with 36 channels (Figure 4c). Four of the 36 contact pads were used to test the electrical conductivity at the manufacturing stage and not used for registration neural activity. Installation of electrodes on the special board was carried out by welding with gold conductors using ultrasonic welding technology. The result of welding the electrodes is shown in Figure 4f. The weight of the multi-electrode array soldered to the board with the connector was 0.675 g. The multi-electrode array was developed to test a wireless electrophysiological complex on an isolated mouse brain. In terms of its implantation, the configuration of the presented multi-electrode array is more suitable for the cerebral cortex, hippocampus and brainstem of rats or larger rodents.

We understand that not all researchers have the opportunity to make their own multi-electrode array; for this reason, we used one of the most common connectors A79027-001 (Omnetics, Minneapolis, MN, USA) to connect commercial MEAs to a wireless module, for example, NeuroNexus.

### 3.2. Wireless Module with Battery

The wireless module is responsible for receiving commands from the base station (data visualization and recording software) and transmitting the data recorded using the MEA. A list of the main components of the wireless module is presented in Table 2.

For the wireless wearable module, we decided to use a 2.4 GHz wireless channel due to the small size of the receiver transmitter and, accordingly, the size of the printed circuit board of the module. As a result, we chose nRF24L01P, a digital receiver transmitter with GFSK modulation, 126 frequency channels, and configurable transmitter power, but its full operation requires a microcontroller. As a microcontroller in the wireless module, it was decided to use the STM32G071GBU6 (4 × 4 × 0.6 mm) due to its size, energy efficiency, and support of the required periphery. The wireless module also consists of an electrophysiology 32-channel amplifier chip with unipolar inputs and the common reference RHD2132, a 36-channel connector for connecting to a 32-channel multi-electrode array A79027-001, a connector for connection to an optical probe A79607-001 and li-ion battery LP301012 (30 mAh 3.7 V).

**Figure 5:**
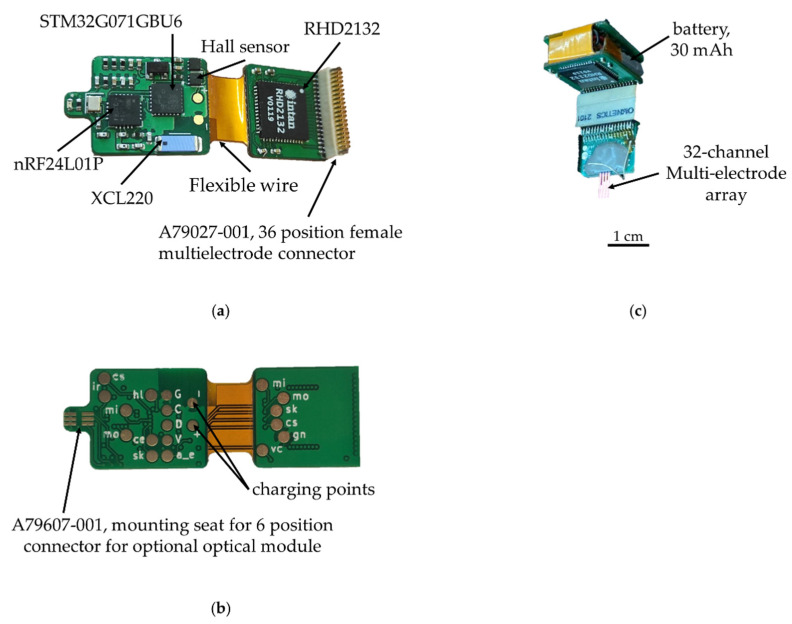
Wireless module of the electrophysiological complex: (**a**) top view, (**b**) bottom view; (**c**) wireless module assembled with battery and multi-electrode array.

The electronic components are placed on a four-layer hybrid rigid-flexible printed circuit board (Figure 5). The assembly of components, with the exception of the connectors, is carried out on one side, which greatly simplifies the preparation process for production and reduces the cost of the final automated assembly. The middle of the board is made in the form of a flexible cable, which allows the board and to be bent and the battery placed in the space between the two halves. This arrangement solves four problems at once: the fastening and placement of the battery, the possibility of the automatic installation of components, the availability of test points for debugging the software part and carrying out automated quality control during assembly, shielding the analog-to-digital converter and analog circuits with the inner layer of the board, and the battery from the radio transmission and digital parts.

**Figure 6:**
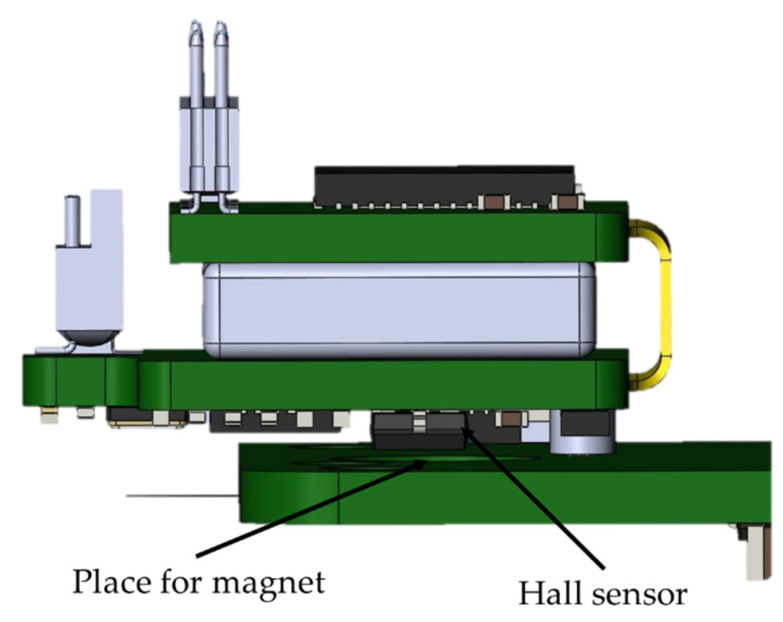
Charging the wireless module in the base station when a magnetic field appears in the Hall sensor.

The battery life of the wireless module is about 3 h. The wireless module is charged only when the wireless module is fixed to the base station. This was achieved through the use of a Hall sensor in the wireless module and a magnet on the PCB of the base station (Figure 6). This solution is used in all modern laptops to determine when to open and close the screen lid. The logic of this work is as follows: when the module is removed from the base station holder, the magnetic field acting on the sensor decreases. The sensor sends a signal to the STM32G071GBU6 microcontroller, which, in turn, starts all peripheral devices and supplies power to the RHD2132 analog-to-digital converter. When the wireless module is installed back into the base station, a magnetic field appears within the range of the Hall sensor, which leads to the shutdown of all peripherals and switches the STM32G071GBU6 microcontroller to an energy-saving mode of operation with a minimum current consumption compared to the self-discharge current of a battery. During charging, the wireless module is in an inactive state, i.e., data from the module are not transmitted. This was achieved through the use of an optical sensor; more details are given in the base station section.

The total weight and dimensions of the wireless module were 2.31 g and 20.31 × 13.39 × 7.95 mm or 20.31 × 13.39 × 12.19 mm considering the connector height, respectively. The weight of the wireless module with the battery and 32-channal multi-electrode array is 2.985 g. This weight is enough for the rodents to freely perform the behavior. For example, the weight of the miniscope is 3.65 g; with this weight, the mouse is able to move around and perform behavioral tests.

### 3.3. Base Station

**Figure 7:**
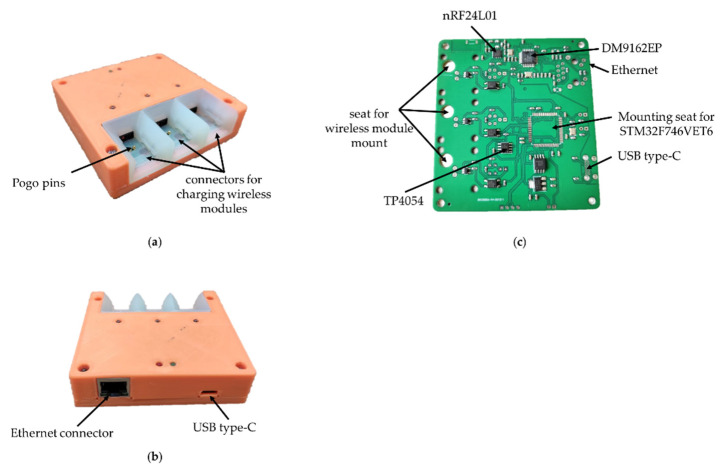
Base station of the electrophysiological complex in 3D printed housing: (**a**) front view; (**b**) back view; (**c**) base station PCB with soldered components.

The base station (Figure 7) is responsible for receiving and transmitting signals from wireless modules for processing data on a PC. In addition, the base station charges the wireless wearable modules. A list of the main components of the wireless module is presented in Table 3.

This is based on the STM32F746VET6 microcontroller, which provides control of the radio part, USB and Ethernet. In addition, this microcontroller implements the protocol for the interaction between wireless modules and a PC. The Ethernet interface is based on the DM9162EP physical layer controller for PC software backward compatibility. For the interaction of the base station with a PC, the USB Type-C interface is used by default due to its prevalence. The current version of USB Type-C has a group of power contacts and the bandwidth of the USB 2.0 standard.

The radio module nRF24L01 is responsible for receiving and transmitting data, the same as in the wireless module. It allows for the simultaneous transmission of signals from three wireless modules due to its bandwidth. Further increases in the number of modules will require the addition of a second radio module, which, in turn, complicates the circuitry and increases the size of the base station PCB.

**Figure 8:**
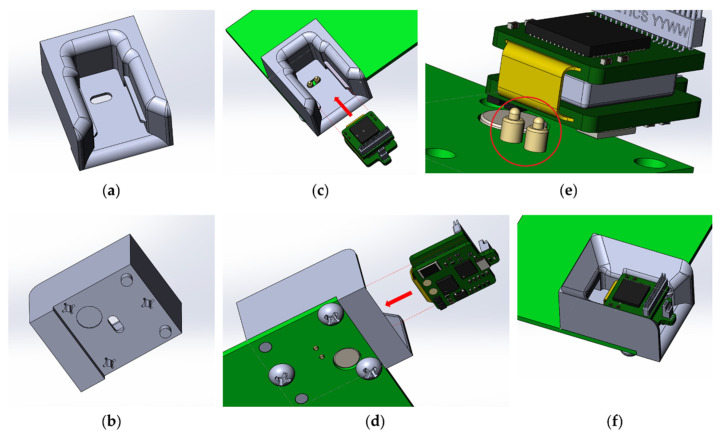
Slot for inserting the wireless module: (**a**) top view, (**b**) bottom view; location of the wireless module for fixing in the slot of the base station: (**c**) top view, (**d**) bottom view, (**e**) dedicated spring loaded pogo pin for charging the wireless module, (**f**) wireless module inserted into the slot of the base station.

The charge controller for Li-ion batteries TP4054 is responsible for charging the wireless modules. The charging time for one wireless module is 30–40 min. To anchor the wireless module in the base station during charging, a plastic mount with guides was developed and printed on an SLA 3D printer (Figure 8). For easier fixation and reliable contact, specialized spring-loaded pogo pin contacts were used as a contact group of the charger.

**Figure 9:**
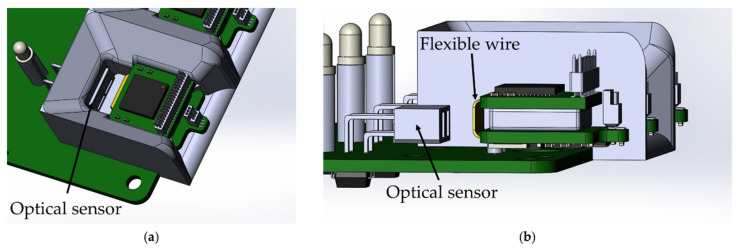
Location of the optical sensor ITR-9909 on the base station board: (**a**) top view, (**b**) side view without showing the wireless module mount.

The base station automatically charges the wireless modules when they are installed in the base station. The function is implemented by using an optical switch ITR-9909, an infrared LED and a phototransistor combined into a single housing. The sensor has maximum sensitivity at a distance of 2.5–5 mm to the object. The location of the optical sensor in the base station was chosen so that reflection of the optical signal occurs from the flexible wire of the wireless module, due to its maximum reflectivity (Figure 9).

This feature allows the base station to determine which modules are currently active and which are not. This is necessary to control the charging process of the wireless module: power is supplied only when the wireless module is installed in the charging mount of the base station and to control the process of polling the modules via the radio channel: data transmission is not carried out from the modules that are being charged.

### 3.4. Data Visualization and Recording Software

**Figure 10:**
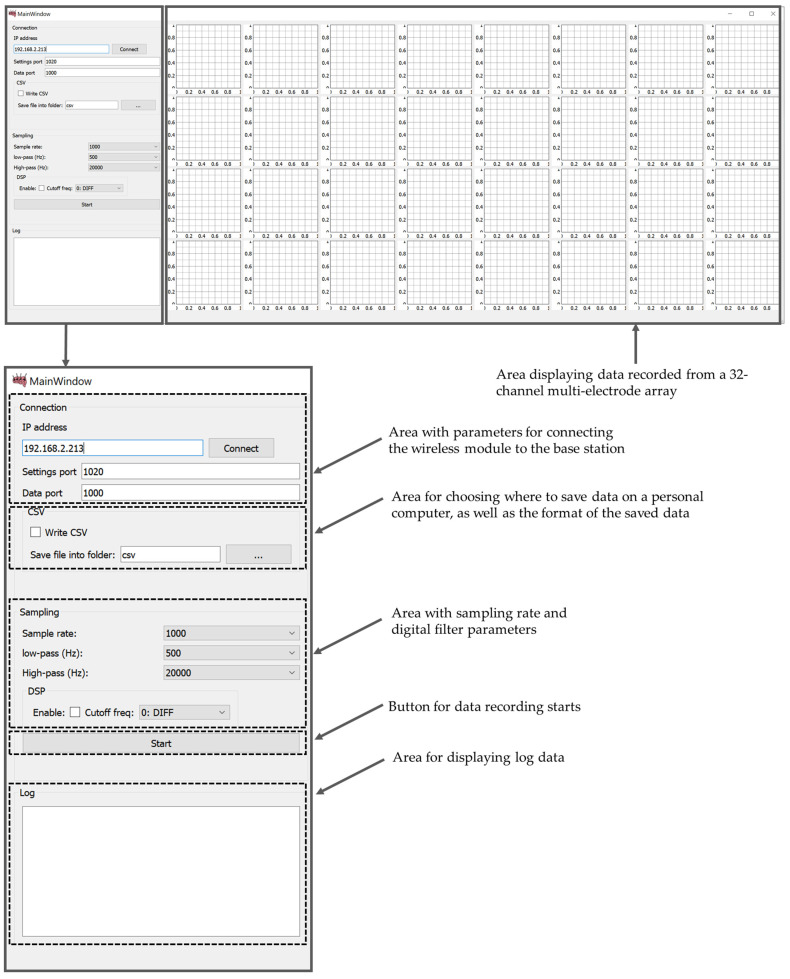
Data visualization and recording software GUI (MouseBrainView, 0.2.3).

The software part (MouseBrainView, 0.2.3, Saint Petersburg, Russia) of the PC is implemented to visualize the signals of 32 measurement channels in real-time and record the information in a CSV file. The main function of data visualization and recording software (current software) is to configure the wireless wearable module and exchange data with it via a local network. The software processes 32 data channels from multi-electrode array, displays the data received from each channel in the form of graphs and records them in a CSV file for further analysis. The software GUI provides the ability to adjust the sampling rate and digital filter parameters for data preprocessing. It can be compiled with a cross-platform bytecode that runs on Windows, Mac OS and Linux. The graphical user interface of the MouseBrainView program is shown in the Figure 10.

In future versions of the data visualization and recording software, we plan to implement support for saving protocols and generating reports on experiments, as well as the ability to quickly send and save data in different formats. We also plan to add:

1. The ability to combine the settings of the selected graphs.
2. The ability to change the names of the axes to units of time and voltage.
3. The ability to display real-time data on the graphs and, in case of packet loss, reflect this in the graph.
4. The ability to account for aliasing when forming graphs (if it is not already taken into account), and preferably smooth the curve.
5. Fourier transform to estimate the signal spectrum for further analysis using third-party tools.

**Figure 11:**
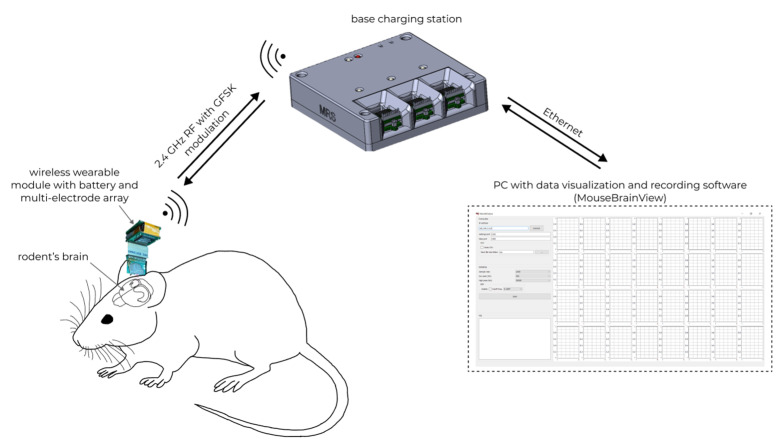
In vivo registration of neural activity in rodents’ brain with wireless electrophysiological complex.

The general scheme of using the WEC is shown in Figure 11.

Neural activity is recorded by a 32-channel multi-electrode array, after which the data are transmitted through the wireless wearable module to the base station using the nRF24L01P via a 2.4 GHz wireless channel. After that, the data received at the base station are sent to the MouseBrainView software installed on the personal computer. Communication between the base station and the personal computer is carried out using Ethernet.

## 4. Conclusions

We have demonstrated the design and fabrication of an open-source wireless electrophysiological complex for in vivo recording neuronal activity, which include wireless wearable modules, base charging station and data visualization and recording software. The main manufacturing steps and one of the possible designs of a 32-channel multi-electrode array were also demonstrated. This complex has a wide potential for use in laboratories conducting electrophysiological studies on the brain. Due to its modern components, wireless connectivity and modular architecture, it can accommodate design changes in the WEC and create more specialized devices. The weight and size of the wireless module with the battery and 32-channal multi-electrode array allow mouse (rodents) to move around and perform behavioral tests. In addition, the WEC is able to register a wide range of signals and has a high noise reduction due to the ability to customize the filters of the recorded signals. The Omnetics connector in the wireless module provides an opportunity to use commercial multi-electrode arrays without having to worry about making one’s own multi-electrode arrays.

In future work, we plan to carry out a registration of neural activity in vivo with a developed and commercial multi-electrode array, to make improvements to WEC. We also plan to carry out a number of modifications to the multi-electrode array, namely to increase the number of registered channels, add the possibility of optical and electrical stimulation, and optimize the size and location of electrical contacts for recording neural activity in the mouse hippocampus. In addition, we are focused on improving the biocompatibility of the presented multi-electrode array. A priority task for us is the use of multichannel electrophysiological registration for miniature fluorescence microscopy.

## Figures and Tables

**Table 1:** In vivo data acquisition systems.

N°	Name	Receiver Size, L × W × H, mm	Receiver Weight (g)	Number of Registration Channels	Measuring the Impedance of the Electrodes	Electrical Stimulation	Light Stimulation	Wireless	Data Transfer Rate (kbit)	Disadvantages Compared to an Open-Source Wireless Electrophysiological Complex
1	Multichannel Systems W2100	13 × 13 × 5.5–15.5 × 15.5 × 6.7	1.9–3.7	4, 8, 16, 32	−	+	+	+	NA	lack of measuring the impedance of the electrodes
2	Jaga Penny	24 × 15.4 × 3	1.2–12	16	−	−	−	+	NA	larger size with a smaller number of registration channels
3	Blackrock Micro Wireless Headstage	35 × 35 × 35	*NA*	Up to 96	−	+	NA	+	NA	lack of measuring the impedance of the electrodes, larger size
4	BioRadio Profi Set. Wireless	100 × 60 × 20	NA	8	−	−	−	+	200	larger size with a smaller number of registration channels, low data transfer rate
5	TaiNi	19 × 14.5 × 15	1.5	Up to 32	−	−	−	+	400	ASIC-based, hard to customize
7	Teleopto Wireless Optogenetics	13 × 18 × 7	1.4	−	−	−	+	+	−	no registration function
8	Neurolux	11.5 × 10.5 × 1.3	0.03	−	−	−	+	+	−	no registration function
9	An open-source wireless electrophysiological complex	10 × 10 × 15	~2	Up to 32	+	in future versions	in future versions	+	1000	

**Table 2:** Main components of the wireless module.

Name	Manufacturer	Description
nRF24L01P	Nordic Semiconductor, Trondheim, Norway	2.4 GHz, 2 Mbit/s, 4 × 4 × 0.8 mm, digital receiver transmitter with GFSK modulation, 126 frequency channels, and configurable transmitter power
STM32G071GBU6	STMicroelectronics, Geneva, Switzerland	4 × 4 × 0.6 mm, microcontroller
RHD2132	Intan Technologies, Los Angeles, CA, USA	an electrophysiology 32-channel amplifier chip with unipolar inputs and common reference
A79027-001	Omnetics, Minneapolis, MN, USA	36-channel connector for connecting to a 32-channel multi-electrode array
A79607-001	Omnetics, Minneapolis, MN, USA	connector for connection to an optical probe
LP301012	Akyga battery, Wroclaw, Poland	30 mAh 3.7 V, li-ion battery

**Table 3:** Main components of the base station.

Name	Manufacturer	Description
nRF24L01P	Nordic Semiconductor, Trondheim, Norway	2.4 GHz, 2 Mbit/s, 4 × 4 × 0.8 mm, digital receiver transmitter with GFSK modulation, 126 frequency channels, and configurable transmitter power
STM32F746VET6	STMicroelectronics, Geneva, Switzerland	microcontroller
DM9162EP	Davicom Semiconductor, Hsinchu, Taiwan	physical layer controller for Ethernet
TP4054	STMicroelectronics, Geneva, Switzerland	charge controller for Li-ion batteries
ITR-9909	Everlight Electronics, Xinbei, Taiwan	an infrared LED and a phototransistor

**Figure A1:**
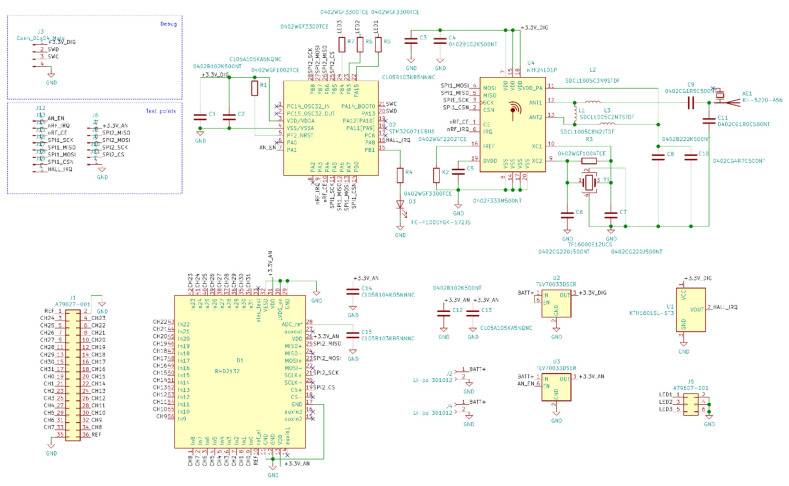
Wireless module electrical schematic diagrams.

**Figure A2:**
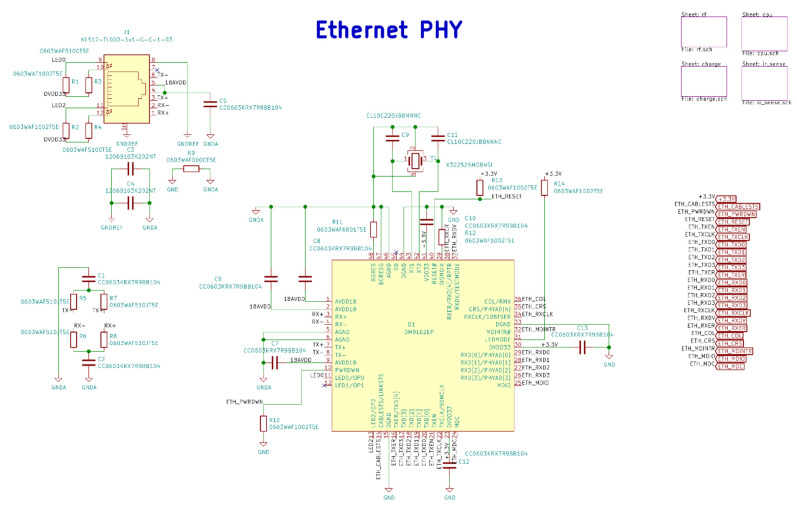
Base station Ethernet PHY electrical schematic diagrams.

**Figure A3:**
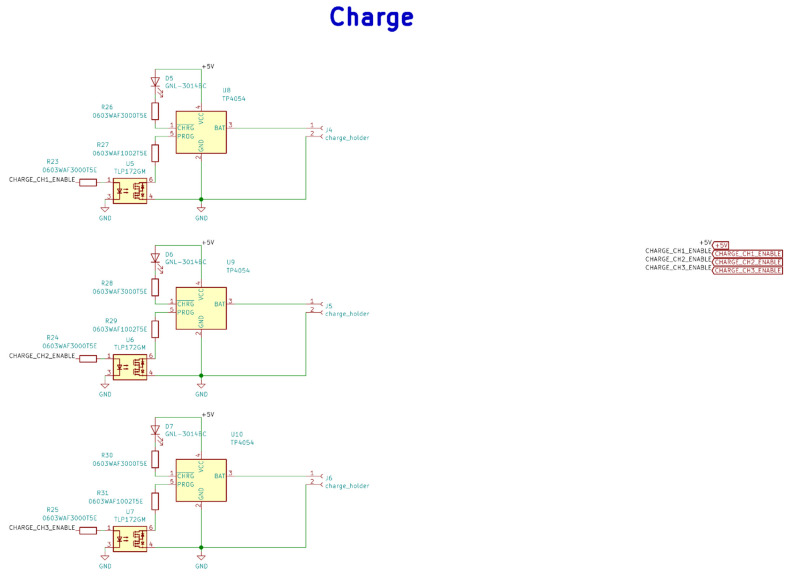
Electrical schematic diagrams of charging in the base station.

**Figure A4:**
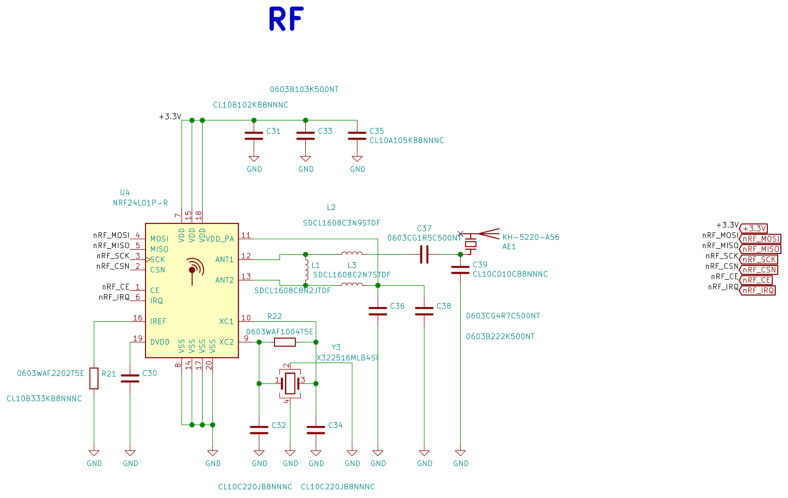
Electrical schematic diagrams of radio frequency system-on-chip in the base station.

**Figure A5:**
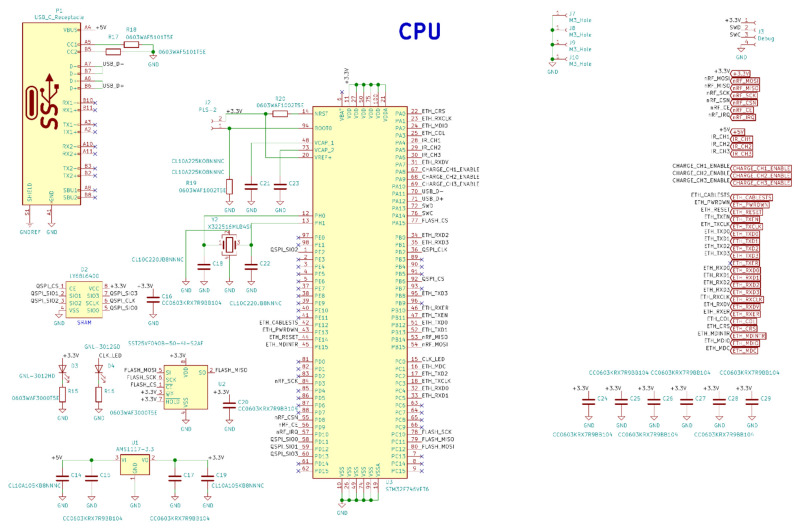
Electrical schematic diagrams of the base station microcontroller and USB Type-C.

**Figure A6:**
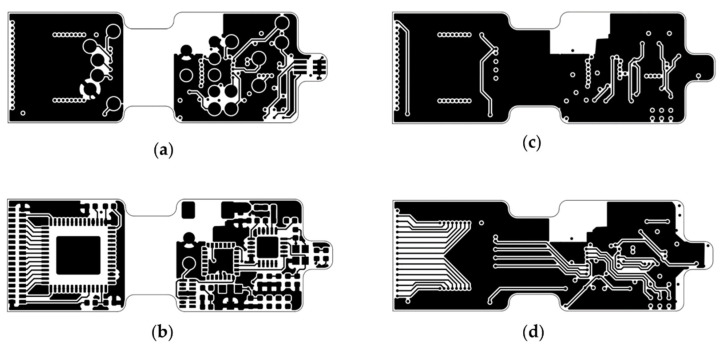
Base station PCBs: (**a**) Layer B_Cu, (**b**) Layer F_Cu, (**c**) Layer In1_Cu, (**d**) Layer In2_Cu.

**Figure A7:**
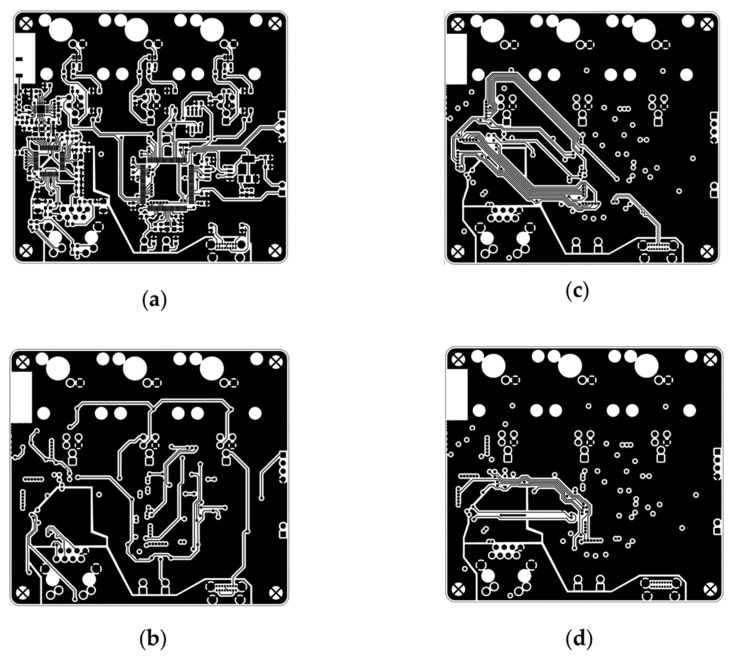
Base station PCBs: (**a**) Layer B_Cu, (**b**) Layer Top_Cu, (**c**) Layer In1_Cu, (**d**) Layer In2_Cu.

## Data Availability

The study did not report any data.

## Appendix A

A schematic diagram and layouts of the printed circuit boards have been shown as Figure A1, Figure A2, Figure A3, Figure A4, Figure A5, Figure A6 and Figure A7.
